# Diseases of the Breast

**Published:** 1903-03-21

**Authors:** 


### HOSPITAL CLINICS AND MEDICAL PROGRESS.

### DISEASES OF THE BREAST.

IN an address given on March 11th before the South-west London Medical Society, Mr. Marmaduke Sheild dealt with some of the more important points in connection with diseases of the breast, and showed some exceedingly fine illustrations of the principal affections of this organ. In his opinion insufficient attention is bestowed, speaking generally, on malformations of the nipple during pregnancy. In those cases where the nipple is insufficiently prominent treatment should be directed to remedying this condition before the birth of the child. The pressure of corsets probably plays an important part in the causation of mal-developed nipple. Eczema of the areola in nursing mothers is a fairly frequent but usually avoidable condition. It must not be confused with Paget's disease. The eczema can be prevented as a rule by the exercise of a little cleanliness. The breast should be washed and dried before and after nursing, and the child's mouth should also receive proper attention. If the baby is suffering from stomatitis this should be treated by the application of glycerine and borax, or, in some cases, of 1 in 4,000 solution of mercury perchloride in glycerine. Primary syphilitic sore is occasionally met with in this region in wet-nurses who have been suckling syphilitic babies; though its characters are sufficiently well marked, its true nature is peculiarly liable to be overlooked. Passing on to diseases of the breast itself, there is one not uncommon affection with which it is often most difficult to deal, namely, neuralgia of the breast. The patient is commonly a pale anæmic spinster or childless wife between the ages of 30 and 40. A very careful examination must be carried out in order to exclude a local cause for the pain. It occasionally happens that some lesion is found such as a small cyst or fibro-adenoma, the removal of which will give relief. In those instances where no such lesion can be found the most successful treatment in Mr. Sheild's experience has been galvanism. Efforts should also be directed towards improving the general health of the patient. In the rare cases of angio-neurotic œdema affecting the breast, where there are recurrent attacks of swelling accompanied by great pain, amputation is indicated, though it is well to be cautious in giving a prognosis, as it sometimes happens that the resulting scar remains the seat of severe neuralgic pain. Hypertrophy of the mammæ may call for amputation if all other methods of treatment are found to be of no avail, and the organs are so large as to seriously incapacitate the patient. Mr. Sheild has had a case under his care of acute hypertrophy in a girl of 21, whose breasts, as seen in the photograph handed round, reached almost to the umbilicus. The condition was successfully treated with constant pressure applied by elastic bandages, together with the internal administration of iodides. It is important to distinguish between true hypertrophy and displacement of the mamma by a large fibro-adenomatous growth, because in the latter case the tumours may be removed without sacrificing the breast. Passing on to an affection of considerable importance to general practitioners—mammary abscess—Mr. Sheild points out that the pathology is now perfectly well understood. It is caused by the entrance of pyogenic organisms into the lymphatics of the skin through some abrasion or fissure of the nipple or areola, and by their passage along these channels to set up suppuration in the deeper structures. The particular microbe most commonly found in the pus from abscesses of the breast is the staphylococcus aureus. The affection occurs most frequently among the more careless and ignorant classes. To illustrate his contention that mammary abscess during lactation is an avoidable affliction he states that at a large lying-in charity in London, among some thousands of women who have been confined in the institution during the last few years, not a single case of mammary abscess has occurred. The early diagnosis of suppuration in the breast is by no means always easy. It is often difficult to avoid confusion between this and "milk congestion." In the latter condition, within a few days of parturition, a part or the whole of a breast may become red, swollen, painful, and acutely tender, and the patient's temperature may be raised to 103° or 104°. For his own part Mr. Sheild never incises a breast unless he can distinguish fluctuation in it. This he considers to be a sound practical rule, as it enables one with certainty to avoid the calamity of incising a breast and failing to find pus, a mistake which he has known to occur on more than one occasion. As soon as fluctuation is recognised the abscess should be opened. The method he recommends is as follows:—A radial incision is made into the abscess cavity and a counter opening is then made at a lower level at the margin of the breast; a drainage tube is passed through the latter incision into the abscess cavity and the preliminary incision is sutured. By this method better drainage is secured, while there is less scarring than there would be if the abscess were drained through the preliminary incision. The opening should in any case be sufficiently large, otherwise a persistent sinus may remain. The tendency to make too small an incision is often due to the practice of opening the abscess without administering an anæsthetic. It is always advisable, unless there is some special contra-indication, to give a general anæsthetic and perform the operation thoroughly. The most satisfactory method of dealing with old neglected cases where the breast is riddled with sinuses consists in freely opening all the sinuses and tamponading with iodoform gauze. Chronic mammary abscess is occasionally met with, and may be tuberculous in origin. It is one of the pitfalls with which the diagnosis of tumours of the breast is beset. The following is a case in point:—An elderly lady consulted an eminent surgeon about a tumour in her breast. There was a hard lump beneath the areola, the nipple was retracted, and the skin showed puckering. A diagnosis of carcinoma was arrived at, and the entire organ was amputated. After removal a section was made through the centre of the breast and the growth, when the "tumour" was found to be a chronic abscess, and the puckering of the skin was due to the scarring and contraction of an old sinus. In such a case diagnosis is quite impossible unless an exploratory incision be made into the tumour previous to its removal. Mr. Sheild most strongly advocates that in all cases this step should be made a routine practice in order to avoid such a calamity as the performance of a complete operation for removal of a cancerous breast, including a dissection of the axilla, only to find afterwards that the disease is not malignant at all. Chronic interstitial mastitis is of interest for two reasons, namely, the difficulty of diagnosing it from scirrhus, and the fact that it gives rise to the formation of cysts. He believes that chronic interstitial mastitis gives rise to the formation of cysts in the breast in a manner similar to that in which chronic interstitial nephritis causes cysts in the kidney. Cysts of the breast are of various kinds, and may in some cases be difficult to diagnose from cancer. Those found in association with old chronic mastitis may be single or multiple, and in the latter case the whole organ may be occupied by numbers of cysts in a fibrous tissue matrix. The treatment of single cysts consists in excision of the cyst and bringing together the deep portions of the wound by means of sutures passed through the skin and deeply through the breast tissue. By this means collection of blood in the deep parts of the wound is avoided and the dangers of sepsis are consequently diminished. Drainage should at the same time be employed. In the case of large single cysts, where excision is inadvisable or is refused by the patient, treatment by tapping and injection is indicated. In any of these cysts intra-cystic growths may be met with, which vary in character in different cases between those of a villous nature and those which are solid fibro-adenomata. When an intracystic growth invades and infiltrates the surrounding breast tissue it becomes a duct cancer. In this case it is advisable to amputate the breast. With regard to the diagnosis of scirrhus of the breast the following points are of importance. The disease is usually painless at first, and is commonly discovered accidentally by the patient; if a small tumour in the breast gives rise to much pain, it is more likely to be a cyst than a cancer. If the lump varies in hardness or in size from time to time this again will favour a diagnosis of mammary cyst. To distinguish between chronic interstitial mastitis and scirrhus Mr. Sheild thinks the following will suffice: On pinching up the growth between the fingers and thumb a definite tumour is felt; if on palpating the breast with the flat of the hand this tumour disappears the case is one of chronic interstitial mastitis. An error that is occasionally made is to mistake a prominent fourth-rib cartilage for a mammary tumour, and this is most likely to occur when a patient seeks advice for neuralgia of the breast. If there are several separate tumours in the breast it is unlikely that the case is one of scirrhus. It is excessively rare for scirrhus to occur otherwise than as a single tumour. The question of whether there are enlarged glands or not in the axilla does not help much. In every case of scirrhus of the breast in which Mr. Sheild has examined the axillary glands they have been found by the microscope to contain deposits of cancer cells, although in many cases they showed no change to the naked eye. If there are large glands of stony hardness in the axilla they of course indicate that the disease is scirrhus. In every case an exploratory incision into the tumour should be made previous to removal of the breast.

Cases unsuitable for operation are those which are too far advanced to offer any chance of complete removal. Belonging to this class are those instances where enlarged glands are felt above the clavicle, where the skin is widely involved, or where the growth is fixed to the ribs. In cancer of outlying lobules of the breast this occurs quite early, as only a layer of fascia intervenes between the growth and the intercostal muscles. It is as well, in Mr. Sheild's opinion, not to operate upon atrophic scirrhus. Patients suffering from this form of cancer may live twenty years or so in comparative comfort, and ultimately die of some other illness, if they are not operated upon. It is also doubtful if it is worth while to operate on cases of carcinoma of the breast occurring in young and pregnant women. It is a terrible disease under these circumstances, growing very rapidly and showing a great tendency to recur shortly after operation if this be undertaken. In those advanced cases where operation does not offer a chance of relief or cure recourse may be had to the *x*-rays. That treatment is in its infancy, and not much can be postulated with regard to the results at present. Mr. Sheild has not seen a case cured by the use of the *x*-rays, though he has seen cases where the course of the disease has been arrested, and in all these the cancer has been superficial, and consisted of recurrent nodules in the skin. Deep-seated and primary growths appear to be almost, or quite, unaffected by the application of the *x*-rays. The only treatment admissible in all cases where the disease is not too far advanced is complete removal by operation. Medical men should be quite firm on this point, since patients will be readily deluded into making a trial of any treatment which appears to offer them the slightest chance of a cure without the necessity of an operation.

